# Does the different photosynthetic pathway of plants affect soil respiration in a subtropical wetland?

**DOI:** 10.1002/ece3.2523

**Published:** 2016-10-13

**Authors:** Jingrui Chen, Qiulin Wang, Ming Li, Fan Liu, Wei Li

**Affiliations:** ^1^ Key Laboratory of Aquatic Botany and Watershed EcologyWuhan Botanical GardenChinese Academy of SciencesWuhanHubeiChina; ^2^ Institute of Soil & Fertilizer and Resources & EnvironmentJiangxi Academy of Agricultural SciencesNanchangJiangxiChina; ^3^ Jiangxi Academy of SciencesNanchangJiangxiChina; ^4^ Hubei Key Laboratory of Wetland Evolution & Ecological RestorationWuhan Botanical GardenChinese Academy of SciencesWuhanHubeiChina

**Keywords:** bacterial respiration, fungal respiration, photosynthetic pathway, soil respiration, substrate‐induced respiration

## Abstract

Plants with different photosynthetic pathways could produce different amounts and types of root exudates and debris which may affect soil respiration rates. Therefore, wetland vegetation succession between plants with different photosynthetic pathways may ultimately influence the wetland carbon budget. The middle and lower reaches of the Yangtze River has the largest floodplain wetland group in China. Tian'e Zhou wetland reserve (29°48'N, 112°33′E) is located in Shishou city, Hubei province and covers about 77.5 square kilometers. Hemathria altissima (C4) was found gradually being replaced by Carex argyi (C3) for several years in this place. An in situ experiment was conducted in Tian'e Zhou wetland to determine the change of soil respiration as the succession proceeds. Soil respiration, substrate‐induced respiration, and bacterial respiration of the C4 species was greater than those of the C3 species, but below‐ground biomass and fungal respiration of the C4 species was less than that of the C3 species. There were no significant differences in above‐ground biomass between the two species. Due to the higher photosynthesis capability, higher soil respiration and lower total plant biomass, we inferred that the C4 species, H. altissima, may transport more photosynthate below‐ground as a substrate for respiration. The photosynthetic pathway of plants might therefore play an important role in regulating soil respiration. As C. argyi replaces H. altissima, the larger plant biomass and lower soil respiration would indicate that the wetland in this area could fix more carbon in the soil than before.

## 1 Introduction

Soil respiration is estimated to be 80 Pg C yr^−1^ and is the second largest flux of carbon between terrestrial ecosystems and the atmosphere (Raich, Potter, & Bhagawati, 2002). The annual carbon (C) flux through soil respiration represents approximately 10% of the atmospheric C pool and is 10 times greater than that through fossil fuel combustion (Raich & Schlesinger, 1992; Schlesinger & Andrews, 2000). Small changes in soil respiration across large scale may affect atmospheric carbon dioxide concentration that is critical for the atmospheric and surface temperature of the earth and ultimately affects global warming (Grace & Rayment, 2000; Schlesinger & Andrews, 2000).

Temperature (soil temperature and/or air temperature) and/or soil moisture were believed to be the key factors in driving soil respiration change in forests (Ekblad, Boström, Holm, & Comstedt, 2005), deserts (Feng et al., 2014), wetlands (Chen et al., 2013), and other ecosystems (Barthel, Cieraad, Zakharova, & Hunt, 2014; Lloyd & Taylor, 1994; Wildung, Garland, & Buschbom, 1975). For example, soil temperature accounted for 70.8% of the variation of soil respiration under natural condition (Chen et al., 2013). Soil respiration mainly includes root respiration and microbial respiration. All these abiotic factors affected soil respiration through their impacts on biotic factors. Photosynthesis is considered as the important process linking the abiotic and biotic factors that affect soil respiration (Barthel et al., 2011; Ekblad et al., 2005; Kuzyakov & Cheng, 2004). Recent research is increasingly reporting that soil respiration is also determined by photosynthesis (Gong et al., 2015; Högberg et al., 2001; Jing et al., 2014; Kuzyakov & Gavrichkova, 2011). Appropriate temperature and soil water content are beneficial to plant growth. The better a plant grows, the more the photosynthate and root biomass can be produced, and the more the photosynthetic products are transported to roots and into soil carbon substrates, and this could contribute to higher root and microbial respiration (Gong et al., 2015; Han, Zhang, Wang, Jiang, & Xia, 2012; Jia et al., 2013).

Previous studies have shown that soil respiration is closely related to recent canopy photosynthesis on time scales ranging from hours to days using phloem girdling (Högberg et al., 2001; Jing et al., 2014), root exclusion by trenching (Gaumont‐Guay, Black, Barr, Jassal, & Nesic, 2008), shading and defoliation (Jing et al., 2014; Kuzyakov & Cheng, 2004), and isotopic labeling (Barthel et al., 2011; Ekblad et al., 2005; Wingate et al., 2010). In grassland, a time lag of <3 hr was found between photosynthetic production and below‐ground respiration (Bahn, Schmitt, Siegwolf, Richter, & Brüggemann, 2009; Yan, Chen, Huang, & Lin, 2011). These short time lags between soil respiration and photosynthesis suggest that soil respiration is closely linked to recent photosynthate (Kuzyakov & Gavrichkova, 2011). Although all this research proved that soil respiration was strongly related to plant photosynthesis, the potential relationship between the photosynthetic pathway of plants and soil respiration has not been explicitly addressed (Schönwitz, Stichler, & Ziegler, 1986).

The middle and lower reaches of the Yangtze River, the largest floodplain in China, have hundreds of wetlands with an average water depth <5 m. Tian'e Zhou wetland, a typical representative of these wetlands located in the middle and lower reaches of Yangtze River, covers about 77.5 square kilometers. It is a seasonal flooded wetland, with *Hemathria altissima* (with the C_4_ photosynthetic pathway) and *Carex argyi* (with the C_3_ photosynthetic pathway) being the dominant plant species. The Three Gorges Dam, completed in 2006, has reduced flood flows downstream in the wet and dry season (Xu & Milliman, 2009) which will lead to lowered groundwater tables. Lowering of the groundwater table has been suggested to be the driving forces for vegetation succession (Lameire, Hermy, & Honnay, 2000). After the completion of Three Gorges Dam, we found that the distribution of *H. altissima* was declining and was gradually being replaced by *C. argyi* (data unpublished), which may relate to hydrologic regime changes downstream. We chose *H. altissima* and *C. argyi* as representative of C_4_ and C_3_ plant and to address: (1) Are there any differences of soil respiration between the two species? If so, could the differences be explained by the photosynthetic pathway, plant productivity, and/or plant biomass; (2) as the succession progressed, will it increase or decrease soil respiration in this area?

## 2 Materials and Methods

### 2.1 Study site

Tian'e Zhou wetland, a natural reserve dedicated to the protection of Pere David' deer, is a periodical flooded marsh located in Shishou city, Hubei province, along the north side of Yangtze River. It has a typical subtropical monsoon climate, and its mean annual temperate and annual precipitation are 16°C and 1200 mm, respectively. *Hemathria altissima* and *C. argyi* were the two dominant species in this wetland although *C. argyi* covers a larger area. *Hemathria altissima* mainly grew 3 m to 500 m from the river, while *C. argyi* grew 0 m to 1000 m from the river (Yang et al., 2011). The two species are distributed in a patchy and miscellaneous pattern in different areas in the wetland depending on soil moisture.

Field experiments were carried out from May 2009 to April 2010. An experimental area (100 × 100 m) was fenced with a metal barrier to prevent disturbance by deer and humans. The C_4_ species and the C_3_ species distributed in the experimental area were mainly in the patchy patterns. As the two species were co‐occurring species in the natural reserve, we assumed that the soil physico‐chemical characteristics, soil temperature, and soil moisture of the two species were the same. Six plots were set up for each species (5 × 5 m) for soil samples and soil respiration measurement. All of the measurements and sample collections were performed monthly.

### 2.2 Soil respiration

Soil respiration was measured from 9:00 a.m. to 11:00 a.m. once every month from May 2009 to April 2010, with a LICOR‐6400 portable photosynthesis system equipped with a LICOR 6400‐09 soil respiration chamber (LICOR, Inc., Lincoln NE, USA). Polyvinyl chloride collars (10.4 cm diameter × 5 cm height) were inserted into the soil to a depth of 1.5 cm at least 24 hr prior to each measurement to reduce a disturbance‐induced CO_2_ efflux (Chen et al., 2013).

### 2.3 Above‐ and below‐ground biomass

Six 1 × 1 m quadrats (spaced more than 10 m) were chosen randomly at each sampling time for each species. All above‐ground plant materials were collected in each quadrat and dried in an oven at 80°C for more than 48 hr and weighed.

In each quadrat, six soil cores (8 cm diameter × 30 cm height) were taken for the measurement of below‐ground biomass (BB). Soil cores were immersed separately in water for more than 24 hr and washed gently through a 60‐mesh sieve. The BB was collected from the sieve then dried at 80°C for more than 48 hr and weighed.

### 2.4 Soil samples and analysis

Soil samples were collected randomly for each species from the surface layer (0–10 cm), the day when soil respiration was measured, and delivered to the laboratory as soon as possible under cooled conditions. They were ground and sifted through a 2‐mm sieve to remove roots and debris. Half of the soil samples were stored at 4°C for substrate‐induced respiration, fungal respiration, and bacterial respiration analysis, and the other half was air‐dried for analysis of soil organic matter and total carbon.

Substrate‐induced respiration, fungal respiration, and bacterial respiration were measured by the incubation method. CO_2_ evolved from the soil (30 g soil per sample) during 24‐hr incubation at 20°C in a 1000‐ml glass bottle was trapped by 3 ml of 0.5 mol L^−1^ NaOH and then quantified by titration with 0.05 mol L^−1^ HCl after addition of excess BaCl_2_. The three kinds of respiration above were expressed as mg CO_2_ g^−1^ (dry weight soil) day^−1^. Blank samples (no soil) were used to assess CO_2_ trapped during incubation (from the air closed in bottle) and handling. Soils for fungal and bacterial respiration were added with following different treatments: (1) substrate‐induced respiration: glucose; (2) fungal respiration: glucose + bactericide (Bronopol); (3) bacterial respiration: glucose + fungicide (Captan). The amounts of glucose, bactericide, and fungicide used were 10 mg g^−1^, 8 mg g^−1^, and 1 mg g^−1^, respectively (Chen et al., 2013). Soil bacterial and fungal densities were measured by plate count methods. Soil organic matter content was measured by the ignition loss method (Dean, 1974). Soil total carbon was measured with Vario Micro cube elemental.

### 2.5 Statistical analysis

All statistical analyses were performed by SPSS 13.0 (SPSS for Windows, version 13.0). Significant differences between the two species were analyzed by repeated‐measures ANOVA (RM‐ANOVA) with sites being the main factor and time being the repeated measure. The data collected over the entire year for each species were treated as a whole for comparison in the RM‐ANOVA. Correlation analysis (soil respiration, above‐ground biomass (AB), BB, substrate‐induced respiration, bacterial respiration, fungal respiration, density of bacteria, and density of fungi) was carried out with the Pearson test. Stepwise regression analysis was applied to explore the importance of different variable on soil respiration. Soil respiration was set as the dependent variable, and AB, BB, fungal respiration, bacterial respiration, and substrate‐induced respiration were the initial independent variables. Backward elimination was used to eliminate the redundant variables. The significance level was set as *p *<* *.05.

## 3 Results

### 3.1 Soil respiration and microbial respiration

**Figure 1:**
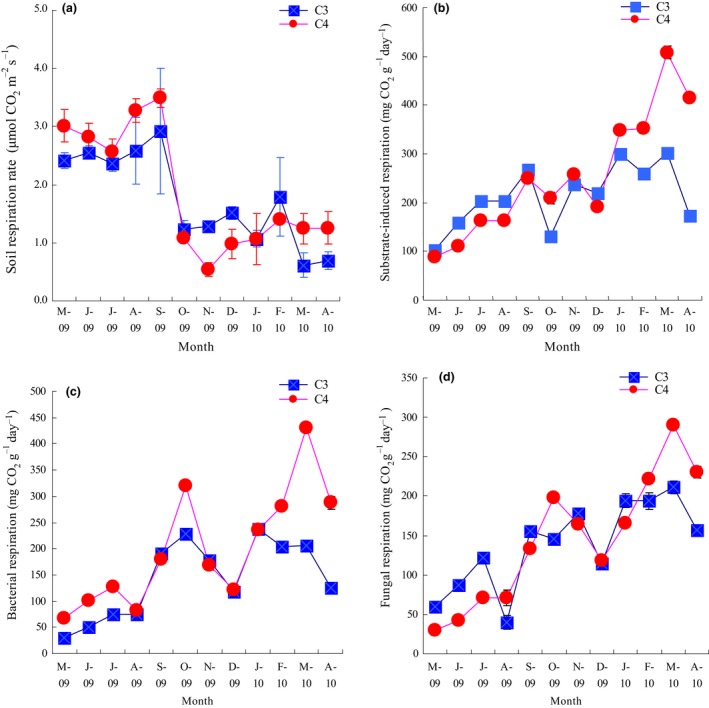
Changes of soil respiration (a), substrate‐induced respiration (b), bacterial respiration (c), and fungal respiration (d) of the C_3_ and C_4_ species. Vertical bars are the standard error of the mean

During the growing season (May to September in 2009), soil respiration rates of the two species were relatively high (Figure 1a), but decreased quickly in October and the low level was maintained until the end of the experiment. The soil respiration of the C_4_ species was significantly larger than that of the C_3_ species over the whole time (RM‐ANOVA, *p *<* *.001). Substrate‐induced respiration of the two species showed the same trend as their soil respiration rate (Figure 1b): From May to September in 2009, both of the C_4_ and C_3_ species' substrate‐induced respiration increased gradually and then decreased in October. The highest values of the C_4_ and C_3_ species' substrate‐induced respiration were 508.3 mg g^−1^ day^−1^ and 301.4 mg g^−1^ day^−1^, and both occurred in March 2014. RM‐ANOVA analysis showed that the C_4_ species had higher substrate‐induced respiration than that of the C_3_ species (*p *<* *.01). In most months of the experiment, the C_4_ species showed higher bacterial respiration than that of C_3_ (Figure 1c), but the fungal respiration showed otherwise (Figure 1d). RM‐ANOVA analysis showed that both bacterial respiration and fungal respiration differed between the two species (*p *<* *.01).

### 3.2 Above‐ground and below‐ground biomass, soil organic matter

**Figure 2:**
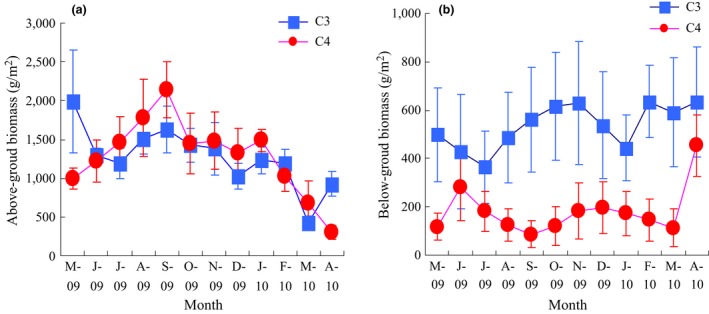
Changes in the above‐ground biomass (a) and below‐ground biomass (b) of the C_3_ and C_4_ species. Vertical bars are the standard error of the mean

**Figure 3:**
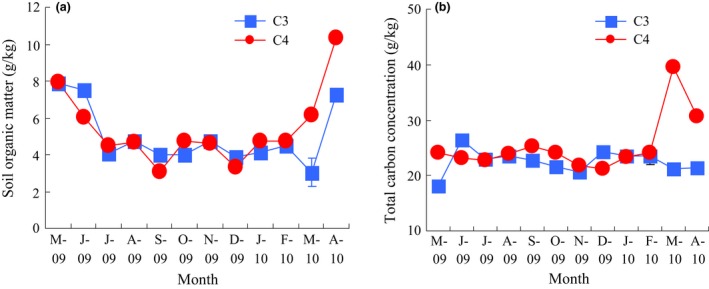
Changes in soil organic matter (a) and soil total carbon concentration (b) of the C_3_ and C_4_ species. Vertical bars are the standard error of the mean

From May 2009, the AB of the C_4_ species increased and reached a peak value of 2139 g m^−2^ in September and then decreased (Figure 2a), while highest value of the C_3_ species, 1993 g m^−2^, occurred in May 2009. However, the AB of the two species was not significantly different (RM‐ANOVA, *p *>* *.05). In contrast, the C_4_ species had a lower BB every month, and RM‐ANOVA analysis also showed that the difference between the two species was significant (*p *<* *.01) (Figure 2b). Soil organic matter and total carbon showed no significant differences between the two species over the whole time (Figure 3a,b).

### 3.3 Relationship between soil respiration and biotic variable

The soil respiration of the two species showed significant relationships with their ABs, bacterial respirations, fungal respirations, and fungal densities (Table 1). Soil respiration of the C_4_ species also showed a significant relationship with density of bacteria while the C_3_ species did not. The AB and BB of the C_4_ species were positively related. Bacterial respiration and fungal respiration were positively related in both species. Stepwise regression analysis showed that annual soil respiration could be expressed by the following equation:

**Table 1:** The Pearson correlation between soil respiration and the seasonal variables during the whole year

		AB	BB	SIR	BR	FR	DB	DF
C_3_	SR	.440*	−.393*	.139	−.566**	−.519**	−.290	.683**
C_4_	SR	.444*	−.114	−.095	−.564**	−.685**	−.373*	.471*

SR, soil respiration; AB, above‐ground biomass; BB, below‐ground biomass; SIR, substrate‐ induced respiration; BR, bacterial respiration; FR, fungal respiration; DB, density of bacteria; DF, density of fungi.

*p <* *.05; **p <* *.01.

$$\begin{array}{cc} \text{Soil respiration}\;(C_{3})= & 3.709-0.008\;\text{FR}\\ & -0.305\;\text{BB}\;(R^{2}=.448,\;t=-2.158,\;p=.038) \end{array}$$

$$\begin{array}{cc} \text{Soil respiration}\;(C_{4})= & 3.106-0.013\;\text{FR}-1.433\;\text{BB}\\ & +0.001\;\text{AB}\;(R^{2}=.728,\;t=3.073,\;p=.002) \end{array}$$

FR: fungal respiration; BB: below‐ground biomass; AB: above‐ground biomass.

## 4 Discussion

### 4.1 Soil respiration and plant photosynthesis

Plant productivity or biomass is believed to be a key driver in regulating soil respiration changes among different plant species, plant species richness, plant physiological genotypes, and plant community composition (Bréchet et al., 2009; Dias, Van Ruijven, & Berendse, 2010; Metcalfe, Fisher, & Wardle, 2011; Migliavacca et al., 2015). Han et al. (2014) showed that changes in gross primary productivity caused by sunny days and adjacent cloudy days regulated the changes in daytime soil respiration under the same soil temperature, and concluded that the effect of photosynthesis of plants on soil respiration should be taken into consideration in order to simulate accurately the magnitude and temporal variation of soil respiration. However, results of some other research showed the opposite (e.g., De Boeck et al., 2007; Johnson, Phoenix, & Grime, 2008). Johnson et al. (2008) reported that the effects of plant community on soil respiration were more than shoot biomass and root density, and suggested that other mechanisms (such as arbuscular mycorrhizal existence) may exist in determining variation in soil respiration. In our study, although both above‐ and below‐ground biomass showed significant relationships with soil respiration (Table 1), the C_3_ species with higher total plant biomass (the sum of above‐ and below‐ground biomass) produced lower soil respiration. The results also revealed that higher plant biomass did not necessarily lead to higher soil respiration, and possibly therefore, there were some other factors involved that counteracted production‐induced stimulation of soil respiration. It was inferred that the photosynthetic pathways of the two species may be one of these other factors.

Root respiration, as an important part of soil respiration, was defined as respiration by roots, their associated mycorrhizal fungi and other microorganisms in the rhizosphere directly dependent on labile C compounds leaked from roots (Nordgren, Ottosson Löfvenius, Högberg, Mellander, & Högberg, 2003). Its carbohydrate sources primarily came from plant photosynthate (Davidson & Holbrook, 2009). Root respiration is very sensitive to changes in photosynthesis and decreased significantly without photosynthesis (Ekblad et al., 2005; Högberg et al., 2001). Root respiration and rhizomicrobial respiration can consume up to 30% of total net photosynthetic production (Cheng et al. 1993). Labeling of photosynthate with ^13^C showed that the contribution of currently assimilated carbon to total root respiration reached constant values of 40%–60% in *Glycine max* (Hansen, Yoneyama, & Kouchi, 1992; Kouchi, Akao, & Yoneyama, 1986). In a previous study, net photosynthetic rate was higher in *H. altissima* (the C_4_ species) than in *Carex cinerascens* (another C_3_ species) in Tian'e Zhou wetland (Li, Yang, & Li, 2007). However, its total biomass was lower than that of the C_3_ species. It has been reported that photosynthesis could affect soil respiration by altering below‐ground substrate availability (Sampson, Janssens, Curiel Yuste, & Ceulemans, 2007). Hence, we inferred that more photosynthate was allocated to below‐ground as available substrate for root and microbial respiration in the C_4_, compared to the C_3_ species.

### 4.2 Microbial activity and soil carbon input

Microbial communities contributing to soil heterotrophic respiration are mainly composed of bacteria and microfungi (Smith & Paul, 1990). In this study, the C_4_ species favored higher bacterial respiration while the C_3_ species had higher fungal respiration. This difference may relate to different quantity and quality of plant litter and root exudation caused by different photosynthetic pathways. Changes in litter quantity and quality can directly and/or indirectly influence microbial community activity, abundance, and composition (Bardgett, Bowman, Kaufmann, & Schmidt, 2005; Carney & Matson, 2005; Curiel et al., 2007; De Deyn, Cornelissen, & Bardgett, 2008; Zak, Holmes, White, Peacock, & Tilman, 2003; Zavaleta & Hulvey, 2007), thereby altering carbon cycling rates, and potentially changing soil respiration. Soil total carbon and soil organic matter sampled from the place where the two species grow were not significantly different over the whole year (Figure 3a,b), while the C_4_ species had higher net photosynthetic rates with lower total plant biomass. One possible explanation for this conflicting result is that the C_4_ species may release greater quantities of labile material to the microbial community through its root system (e.g., fine root turnover and exudation), stimulating carbon mineralization in the rooting zone (Baer, Kitchen, Blair, & Rice, 2002), and the higher substrate‐induced respiration in the C_4_ species is consistent with this explanation.

C‐rich substrates, such as sugars (50%–70% of total exudate), carboxylic acids (20%–30% of total exudate), and amino acids (10%–20% of total exudate), make up the majority of exudate compounds (Hütsch, Augustin, & Merbach, 2002; Jones, 1998; Kraffczyk, Trolldenier, & Beringer, 1984). Labile soil C inputs could affect the activity and relative abundance of fungi and bacteria. Mahaney, Smemo, and Gross (2008) showed that C_4_‐derived materials were more abundant in the labile components of active soil organic carbon, while the more stable components of active soil organic carbon were biased toward C_3_‐derived materials. In general, bacterial decomposition pathways support high turnover rates of easily available substrates, while slower fungal‐dominated decomposition pathways favored more complex organic materials (Wardle, Bonner, & Barker, 2002). This is consistent with our study that found higher bacterial respiration rates in the soil below C_4_ species and higher fungal respiration in the soil below C_3_ species. Although the soil below C_4_ species favored higher bacterial respiration, stepwise regression analysis showed that fungal respiration played an important role in soil respiration changes of the two species. The results were consistent with Anderson and Domsch (1973, 1975) that fungal respiration contributed higher percentage on soil respiration than that of bacterial respiration in soils.

### 4.3 Vegetation succession and hydrologic change

Hydrology is a major determinant of wetland vegetation patterns and physiochemical characteristics of wetlands, such as nutrient cycling, organic matter accumulation, and soil chemistry (Mitsch & Gosselink, 2000). Water table is the primary factor underlying the observed vegetation gradient (Zampella, Moore, & Good, 1992), and vegetation succession in wetlands has mostly been related to the change of water table (Elmore, Mustard, & Manning, 2003; Str__mberg, Tiller, & Richter, 1996).

Increased temperatures lead to a lowered groundwater table in many areas (Bouraoui, Vachaud, Li, Le Treut, & Chen, 1999; Brouyère, Carabin, & Dassargues, 2004; Woldeamlak, Batelaan, & De Smedt, 2007), and the decline of the groundwater table will be accelerated by dam construction (Huang, Sun, & Jiang, 2011; Xu & Milliman, 2009). The Three Gorges Dam, completed in 2006, has reduced flood flows downstream in the wet and dry season (Xu & Milliman, 2009), which significantly decreased the water table in downstream lakes and wetlands (Huang et al., 2011). As the Tian'e Zhou wetland lies on the Yangtze River, its water table was inevitably affected by the construction of the Three Gorges dam. *Carex argyi* is distributed in a wider area than *H. altissima* (Yang et al., 2011), which suggests it can tolerate a greater range of ecological conditions. As the water table draws down, *C. argyi* gradually replaces *H. altissima* in this area, causing more carbon to be fixed and less CO_2_ to be released in this area.

## 5 Conclusion

Two dominant species of Tian'e Zhou wetland with different photosynthetic pathways (C_4_ and C_3_) were studied. The results showed that the C_3_ species with higher total biomass produced lower soil respiration, while the C_4_ species with lower total biomass produced higher soil respiration, which was inconsistent with previous studies that high productivity or biomass leads to high soil respiration. It was inferred that photosynthetic pathways may play an important role in determining soil respiration variation between the two species.

## Conflict of Interest

None declared.
